# Microglia-Derived Insulin-like Growth Factor 1 Is Critical for Neurodevelopment

**DOI:** 10.3390/cells13020184

**Published:** 2024-01-18

**Authors:** Dominika Rusin, Lejla Vahl Becirovic, Gabriela Lyszczarz, Martin Krueger, Anouk Benmamar-Badel, Cecilie Vad Mathiesen, Eydís Sigurðardóttir Schiöth, Kate Lykke Lambertsen, Agnieszka Wlodarczyk

**Affiliations:** 1 Department of Neurobiology Research, Institute of Molecular Medicine, University of Southern Denmark, Campusvej 55, 5230 Odense M, Denmark; 2 Institute for Anatomy, University of Leipzig, 04103 Leipzig, Germany; 3 Neuroscience Academy Denmark, Blegdamsvej 3B, 2200 Copenhagen N, Denmark; 4 Department of Clinical Research, BRIDGE—Brain Research Interdisciplinary Guided Excellence, University of Southern Denmark, Campusvej 55, 5230 Odense M, Denmark; 5 Department of Neurology, Odense University Hospital, 5000 Odense C, Denmark

**Keywords:** microglia, neonatal microglia, *Igf1*, neurodevelopment, neophobia, primary myelination

## Abstract

Insulin-like growth factor 1 (IGF-1) is a peptide hormone essential for the proper development and growth of the organism, as a complete knockout of *Igf1* in mice is lethal, causing microcephaly, growth retardation and the defective development of organs. In the central nervous system, neurons and glia have been reported to express *Igf1*, but their relative importance for postnatal development has not yet been fully defined. In order to address this, here, we obtained mice with a microglia-specific inducible conditional knockout of *Igf1*. We show that the deficiency in microglial *Igf1*, starting in the first postnatal week, leads to body and brain growth retardation, severely impaired myelination, changes in microglia numbers, and behavioral abnormalities. These results emphasize the importance of microglial-derived *Igf1* for brain development and function and open new perspectives for the investigation of the role of microglial-*Igf1* in neurological diseases.

## 1. Introduction

Microglia, the resident macrophages of the central nervous system (CNS), play a pivotal role in maintaining brain homeostasis [1,2]. Originating from the yolk sac [3], they colonize the CNS during neurodevelopment, exhibiting a dynamic nature characterized by morphological and functional changes throughout their lifespan. Traditionally, microglia are associated with immune-related functions within the brain and actively patrol the CNS, engaging in debris clearance and rapid response to danger signals. Extensive evidence has emphasized the crucial role of microglia in neurodevelopment [4]. Notably, microglial involvement in synaptic pruning, a critical process during both development and adult life, holds particular significance. Through the phagocytosis of dysfunctional synapses, microglia regulate synaptic numbers, exerting an influence over the structural and functional organization of the brain [5]. Furthermore, our previous studies underscored the vital role of microglia in shaping the developing CNS through the expression of factors that guide the maturation of astrocytes, oligodendrocytes, and neurons [6]. Dysfunctions in microglial activity have been implicated in synaptic and developmental alterations, potentially leading to the onset of neuropsychiatric diseases [7].

During postnatal development, there is a robust upregulation of the gene encoding insulin-like growth factor 1 (IGF-1) in microglia [6]. This growth factor plays a critical role in regulating the growth of brain tissue, controlling neurons and oligodendrocyte populations [8], and supporting the survival of cortical neurons [9]. Rare human cases of IGF-1 deficiency are characterized by growth alteration, microcephaly, sensorineural deafness, and delayed psychomotor development [10]. In mice, the deficiency of *Igf1* and its receptor often leads to postnatal lethality, and mice that do survive to adulthood show microcephaly associated with increased neuronal death and the impairment of myelination [11]. IGF-1 is primarily produced in the liver under the regulation of the pituitary secretion of growth hormones (GHs) [12]. Apart from systemic expression, IGF-1 is also produced locally, including in the brain, with its peak expression during peri- and early postnatal periods [13]. Although microglia are the major source of IGF-1 during postnatal development [6], so far, little is known about the importance of microglial IGF-1 for proper brain development and brain function.

In this study, we utilized tamoxifen-inducible genetic recombination in *CX3CR1*^CreER/WT^: *Igf1*^flox/flox^ mice to selectively eliminate the expression of *Igf1* in microglia. Notably, the microglia-specific deletion of *Igf1* during the first postnatal week had profound effects, leading to a significant decrease in both body and brain growth and ultimately resulting in adult animals exhibiting anxiety and neophobia. Moreover, the absence of microglial *Igf1* led to hypomyelination and increased microglia density. Altogether, our study sheds light on the pivotal involvement of microglia-derived *Igf1* on microglial function and highlights its significance in shaping postnatal development.

## 2. Materials and Methods

### 2.1. Animals

**Figure 1:**
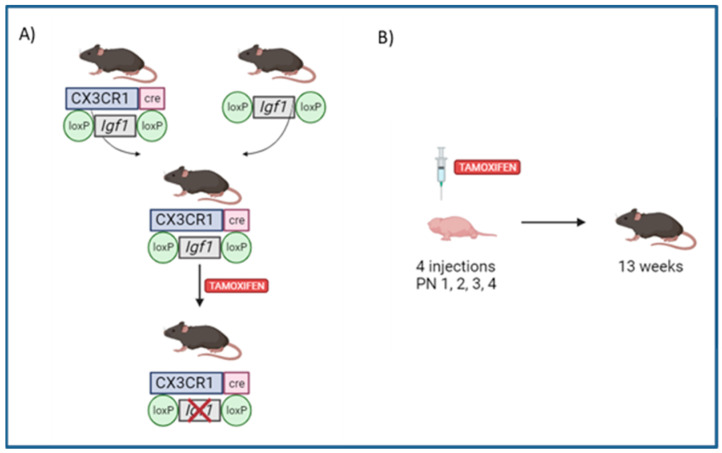
Conditional microglial *Igf1* knockout. (**A**) Illustration of the generation of *CX3CR1*^CreER/WT^: *Igf1*^flox/flox^ (MG-*Igf1*^KO^) strain. (**B**) Illustration presenting the cre recombinase activation. Figure was created in Biorender.

*CX3CR1*^CreER^ [14] and *Igf1*^flox^ [15] were obtained from The Jackson Laboratory and maintained as a breeding colony in the Biomedical Laboratory, University of Southern Denmark (Odense, Denmark), to obtain *CX3CR1*^CreER/WT^: *Igf1*^flox/flox^ (MG-*Igf1*^KO^) (Figure 1A). Cre recombinase was activated by the daily subcutaneous (s.c.) administration of Tamoxifen (TAM) (Sigma Aldrich, St. Louis, MO, USA, 75 mg/kg) to newborn pups from postnatal day 1 to postnatal day 4 (PN1-PN4) (Figure 1B). For control purposes, *CX3CR1*^CreER/WT^ mice (*Igf1*^WT^) were also injected with TAM. This control setup was critical to ensure that any observed effects in the MG-*Igf1*^KO^ group were attributable specifically to the absence of *Igf1* rather than to the previously reported effects of TAM administration [16]. Animal experiments were conducted on female mice in their adulthood (13 weeks old) and were approved by the Danish Animal Experiments Inspectorate (approval number: 2020-15-0201-0065).

### 2.2. Behavioral Tests

Behavioral tests, including open field (locomotor activity), novel object recognition (memory and ability to learn), and marble burying (anxiety and obsessive-compulsive-disorder-like behavior), were performed on 13-week-old female mice.

### 2.3. Open Field

Each mouse was placed in the center of a 45 × 45 × 45 cm non-transparent plastic box for 10 min. Locomotor activity was tracked and recorded by the SMART video tracking system version 3.0 (Panlab, Barcelona, Spain) together with a video camera (SSC-DC378P, Biosite, Stockholm, Sweden) [17]. The incidence or duration of different types of behavior, such as grooming, center and wall rearing, and the presence of urine or fecal boli, was recorded and analyzed.

### 2.4. Novel Object Recognition (Nor)

The open field test, as stated above, was considered as the habituation day (day one) for the NOR test. During the second day of the test, the mouse was placed in a familiar box with two identical objects that were set in equal distance. One of the objects was replaced by the novel one on the third day. In this test, we observed and evaluated the time the mouse spent on discovering items during the last two days of the experiment and calculated the discrimination index to assess the short-term memory in tested animals. The discrimination index indicates the preference of the mouse to the object, varying between +1 and −1, where a positive value corresponds to the time spent exploring the novel object, a negative value corresponds to the time spent exploring the familiar object, and a zero value indicates no preference [18]. The behaviors were recorded by a camera (SSC-DC378P, Biosite, Stockholm, Sweden).

### 2.5. Marble Burying

Standard cages filled with new bedding material to a depth of 5 cm were prepared with 15 marbles distributed on top in equal distances. Each cage was photographed before and after the experiment. Mice were left for 30 min in the cages, and there was one mouse per cage. The number of buried marbles was calculated at the end; only if 2/3 of the marble or more was covered with bedding, it was considered to be buried.

### 2.6. qRT-PCR

Mice were sacrificed via overdose with sodium pentobarbital (Euthanimal, 200 mg/kg of body weight, Glostrup sygehusapotek, Glostrup, Denmark) and perfusion using ice-cold PBS. Next, brain tissue was collected. RNA was extracted from left brain hemispheres using TRIzol (Invitrogen, Waltham, MA, USA) according to the manufacturer’s protocol. cDNA was transcribed from 1 µg of total RNA using the M-MLV reverse transcriptase (Invitrogen) following the manufacturer’s instructions. Quantitative real-time polymerase chain reaction (qPCR) was performed using 1 µL of cDNA combined with 24 µL of Maxima probe/ROX qPCR master mix (Fermentas, Carlsbad, CA, USA) with the TaqMan™ Gene Expression Assay (Applied Biosystem, Waltham, MA, USA) (Table 1). Plates were run on a qPCR machine (QuantStudio 3—Real-Time PCR System, Applied Biosystems, Waltham, MA, USA).

### 2.7. Immunohistochemistry

Right brain hemispheres from pentobarbital anesthetized and PBS-perfused mice were 4% PFA-fixed and cryoprotected in 30% sucrose and then frozen. Following this, twelve-micrometer sagittal sections were cut on a cryostat at −20 °C and stored on Superfrost Plus slides (Thermo Scientific, Waltham, MA, USA). Slides were rinsed in 1 × PBS three times and incubated for 30 min in methanol with H_2_O_2_ to block endogenous peroxidase. After the repeated rinsing of slides in 0.2% Triton X-100 in PBS (PBST), slides were incubated in 3% bovine serum albumin (BSA) in PBST for 30 min to block unspecific binding. Brain sections were incubated with primary antibodies rabbit anti-IBA-1 (Lot: WTF4691, Nordic Biolabs AB, Täby, Sweden, 1:500) and rabbit anti-glial fibrillary acidic protein (GFAP, Lot: 41343165, Agilent technologies, Santa Clara, CA, USA, 1:500) in PBST with 3% BSA for 1 h; rinsed in PBST with 3% BSA, followed by incubation with the secondary antibody biotinylated goat anti-rabbit IgG (Lot: Lot: ab64256, Abcam, Cambridge, UK, 1:2) for 1 h; and rinsed thereafter. Next, the sections were incubated with horseradish peroxidase-conjugated streptavidin (Lot: 17094891, Amersham^TM^, Piscataway, NJ, USA, 1:200) in PBST with 3% BSA for 1 h and rinsed, and staining was developed in 3,3′-Diaminobenzidine (DAB) for 8 min. Sides were then rinsed, dehydrated, mounted, and visualized on an Olympus CX41 microscope.

### 2.8. Stereology

A design-based unbiased stereology approach was used to quantify microglia in the cortex [19]. Cell counting was performed blindly. An Olympus BX 50 microscope (Olympus, Hamburg, Germany) attached to a U-PMTVC Japan color camera (Olympus, Germany) and a three-axis Proscan Prior motorized stage was used. The software Visiopharm Integrator System (Version 3.6.5.0, Visiopharm, Hørsholm, Denmark) was used for cell counting. The region of interest was outlined using a 4× objective (Olympus UplanFl, Tokyo, Japan) according to the cortical area defined in Allen Mouse Brain Atlas (atlas.brain-map.org). Cells were counted using a 100× oil-objective lens (Olympus UPlanFl, Tokyo, Japan). Cells were only counted if their soma touched the inclusion border and did not touch the exclusion border of the counting frame. Cells were excluded if located on the edge of the tissue. In total, 3–6 sagittal sections were counted per animal. The relative number of cortical microglia was estimated using the optical fractionator method and by dividing the number of microglia by the number of counting frames in each section. The sampling grid was applied to the sections, with a step length of 362.17 µm × 272.69 µm. For each region, a counting frame size of (36.22 µm × 27.27 µm) was used with a guard zone of 1 µm and an optical dissector height of 7 µm.

### 2.9. Electron Microscopy

Animals were sacrificed and transcardially perfused with PBS, followed by a fixative containing 4% paraformaldehyde and 1.5% glutaraldehyde. After the postfixation of brains in the same fixative, sagittal sections were acquired using a vibrating microtome (Leica Microsystems, Wetzlar, Germany) with a thickness of 75 μm. The sections were stained with 0.5% osmium tetroxide in PBS for 30 min and subsequently rinsed in PBS followed by dehydration with 30, 50, and 70% ethanol. Afterward, the sections were treated with 1% uranyl acetate in 70% ethanol for 1 h before the tissue was further dehydrated using 80, 90, 96, and 100% ethanol and finally propylene oxide. The sections were transferred in Durcupan (Sigma Aldrich, Steinheim, Germany) and embedded in between coated microscope slides and cover slips before the polymerization process at 56 °C for 48 h. Regions of interest were located via light microscopy, marked, and transferred on blocks of resin for a second polymerization step. Finally, the embedded tissue was cut into semi-thin sections, and areas for ultrastructural analysis were cut into 55 nm ultra-thin sections using an ultra-microtome (Leica Microsystems, Wetzlar, Germany) and transferred on Formvar-coated grids and stained with lead citrate for 6 min. The analysis was performed using a Zeiss SIGMA electron microscope (Zeiss NTS, Oberkochen, Germany). G-ratios of transversally sectioned axons were calculated using ImageJ 1.54h software and compared between groups. Each group consisted of 4 animals (n = 4). Per animal, 130–145 axons from five different regions of the corpus callosum were analyzed. The analysis was performed by an investigator who was blinded to the experiment.

### 2.10. Magnetic-Activated Cell Sorting (MACS)

PN5 mice were sacrificed via pentobarbital overdose, and brains were collected. Brain tissue was forced through a 70 µm strainer (BD Biosciences, Kongens Lyngby, Denmark) in order to obtain a single cell suspension. The sample was centrifuged with 37% Percoll (Cytiva, Marlborough, MA, USA, 17-0891-02), and mononuclear cells were collected. Microglia cells were isolated via magnetic separation using CD11b MicroBeads (Miltenyi Biotec, Gaithersburg, MD, USA). All steps were carried out following the manufacturer’s protocol (Miltenyi Biotec, Gaithersburg, MD, USA).

### 2.11. Statistical Analysis

Statistical analysis was performed using GraphPad Prism 10.1.2 software. All data were assessed for normality distribution. The Shapiro–Wilk, Anderson–Darling, D’Agostino and Pearson, and Kolmogorov–Smirnov tests were used to assess the normality for samples. An unpaired *t*-test with Welch’s correction was used to analyze normally distributed data that passed normality tests. Data that were not normally distributed were analyzed via a Mann–Whitney U nonparametric test. Results were considered significant when *p* < 0.05. All data are presented as the mean ± standard error of the mean (SEM) unless specified otherwise. Asterisks indicate significant differences (* *p* < 0.05; ** *p* < 0.01; *** *p* < 0.001; **** *p* < 0.0001).

## 3. Results

### 3.1. Postnatal Deficiency of Microglia-Derived Igf1 Leads to Growth Alterations

**Figure 2:**
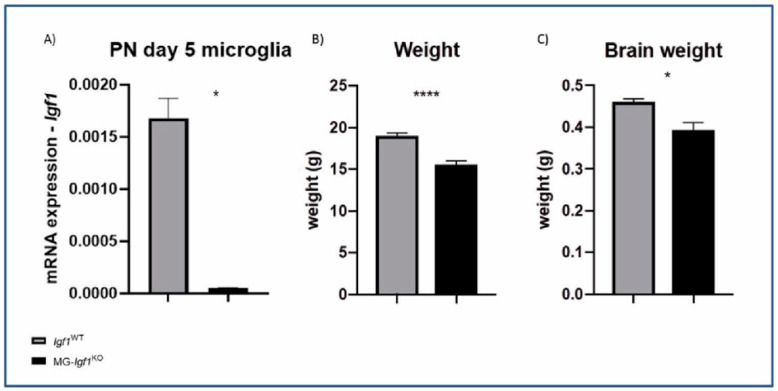
Impact of *Igf1* depletion in microglia on somatic and brain weights. (**A**) Bar graphs showing mRNA expression of *Igf1* in sorted PN5 microglia from MG-*Igf1*^KO^ mice compared to *Igf1*^WT^ controls. Here, 18S was used to normalize data. (**B**) Bar graph illustrating weight (g) of MG-*Igf1*^KO^ (n = 6) and *Igf1*^WT^ (n = 14) mice. (**C**) Bar graph illustrating brain weight (g) of MG-*Igf1*^KO^ (n = 5) and *Igf1*^WT^ (n = 10) mice. For comparison of the two groups, unpaired *t*-test with Welch’s correlation was used for normally distributed data, and the Mann–Whitney U nonparametric test was used for not normally distributed data. Data are represented as mean ± SEM. Asterisks indicate significant differences (* *p* < 0.05; **** *p* < 0.0001).

**Figure 3:**
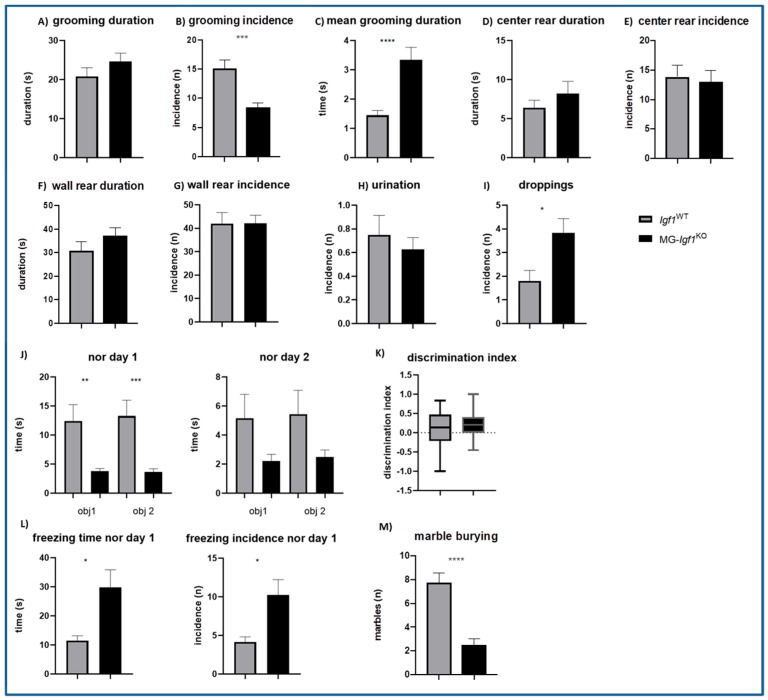
Impact of *Igf1* deficiency in microglia on behavioral outcome. (**A**–**I**) Bar graphs showing behavioral outcomes from open field tests, including grooming duration, incidence, mean grooming duration, center rear, wall rear, presence of urine, and number of droppings in 13-week-old MG-*Igf1*^KO^ (n = 24) and *Igf1*^WT^ (n = 15) mice. (**J**,**K**) Bar graphs showing behavioral outcomes from the novel object recognition test performed on 13-week-old MG-*Igf1*^KO^ (n = 24) and *Igf1*^WT^ (n = 15) mice. (**L**) Bar graphs showing freezing behavior, which is represented as the duration and number of incidences of the behavior observed during the novel object recognition test performed on 13-week-old MG-*Igf1*^KO^ (n = 7) and *Igf1*^WT^ (n = 8) mice. (**M**) Bar graph showing outcomes from the marble-burying test performed on 13-week-old MG-*Igf1*^KO^ (n = 12) and *Igf1*^WT^ (n = 15) mice. For normally distributed data, an unpaired *t*-test with Welch’s correlation was used, and a Mann–Whitney U nonparametric test was used for non-normally distributed data. Data are represented as mean ± SEM. Asterisks indicate significant differences (* *p* < 0.05; ** *p* < 0.01; *** *p* < 0.001; **** *p* < 0.0001).

In order to assess the role of microglial *Igf1* expression during postnatal development, we have generated microglia-specific *Igf1*-deficient animals by crossing *CX3CR1*^CreER^ mice [14] with a transgenic mouse carrying loxP sites flanking exon 4 of the *Igf1* gene [15]. The resulting offspring *CX3CR1*^CreER/WT^:*Igf1*^fl/fl^ (MG-*Igf1*^KO^ here from) and *CX3CR1*^CreER^ control animals (*Igf1*^WT^ here from) were injected with tamoxifen at postnatal days 1–4 to induce cre recombination. This led to *Igf1* depletion, specifically during the peak of microglial *Igf1* expression, which occurs in the first postnatal week [6]. We achieved a ca. 97% reduction in *Igf1* expression in microglia at PN5 (Figure 2A). Although the mice remained viable, they exhibited significant growth retardation, as evidenced by reduced body and brain weight (Figure 2B,C), along with altered behavior in adulthood (Figure 3).

### 3.2. Postnatal Deficiency of Microglia-Derived Igf1 Leads to Increased Anxiety and Neophobia

In order to characterize the impact of microglial *Igf1* deficiency on mice behavior, various behavioral tests were performed on adult animals; open field, novel object recognition, and marble-burying tests were carried out to investigate locomotor activity and anxiety, memory, and stereotypical behavior, respectively [20,21,22].

In the open field test, we focused on the incidence and duration of behaviors, such as grooming, center rearing, and wall rearing; additionally, we noted the number of fecal boli and the presence of urine (Figure 3A–I). A significant difference was detected in grooming, which is a typical behavior in mice characterized by sequenced movements around the face or body, such as scratching, licking, or nibbling [23]. MG-*Igf1*^KO^ mice showed a significantly lower grooming incidence (Figure 3B), while the total grooming duration remained almost unchanged (Figure 3A), resulting in an increase in the mean duration of a single grooming incident (Figure 3C), altogether indicating repetitive behavior [22]. We did not observe any differences in center and wall rearing incidences or duration, nor in urination (Figure 3D–H). Nevertheless, the number of collected fecal boli (Figure 3I) was significantly higher in the MG-*Igf1*^KO^ group, suggesting an increase in the level of anxiety.

The novel object recognition test is based on the innate urge of rodents to explore new objects, allowing us to assess memory and explorative behavior [24]. Both experimental groups showed no preference for exploring novel objects during the test (Figure 3J,K), indicating tamoxifen-induced deficits in short-term memory, as previously described [25]. Interestingly, MG-*Igf1*^KO^ mice were significantly less interested in exploring the objects than the control group (Figure 3J), often presenting freezing behavior (Figure 3L) that is indicative of pronounced anxiety. Next, we used the marble-burying test to assess an obsessive compulsive and anxiety-like behavior in mice. We noticed that MG-*Igf1*^KO^ mice were showing no interest in unfamiliar objects, which resulted in significantly fewer buried marbles (Figure 3M).

### 3.3. Postnatal Deficiency of Microglia-Derived Igf1 Leads to Increased Numbers of Microglia

**Figure 4:**
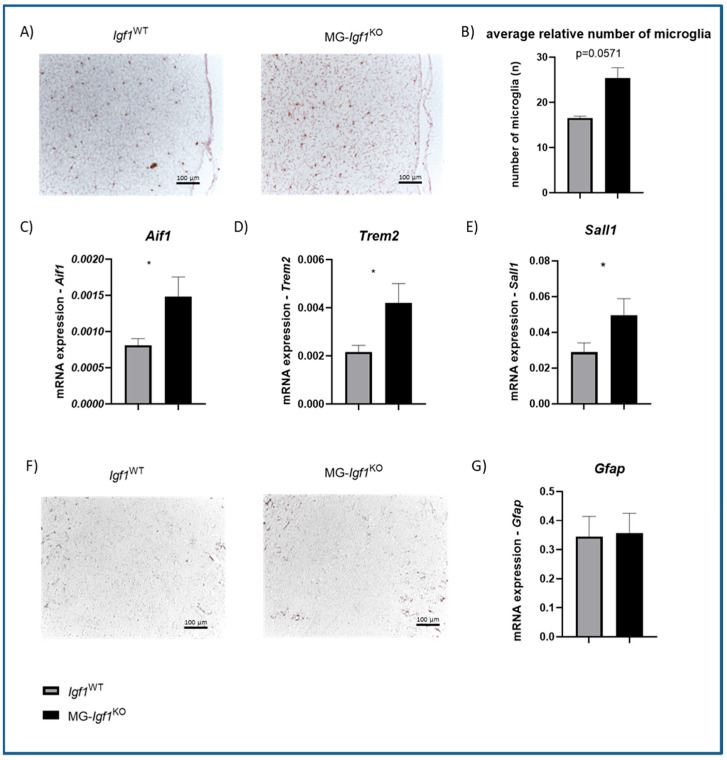
Impact of microglial *Igf1* deficiency on microglia and astrocytes. (**A**) Representative micrographs showing IBA-1 staining of the cerebral cortex from MG-*Igf1*^KO^ mice and *Igf1*^WT^ controls. Pictures were taken with x20 magnification. (**B**) Bar graph showing the average relative number of microglia in the cerebral cortex of MG-*Igf1*^KO^ (n = 3) and *Igf1*^WT^ (n = 4) mice quantified via stereology. (**C**–**E**) Bar graphs showing the mRNA expression of microglia-related genes *Aif1*, *Trem2*, and *Sall1* in brains of MG-*Igf1*^KO^ (n = 10) and *Igf1*^WT^ mice (n = 10). (**F**) Representative micrographs showing GFAP staining of the cerebral cortex from MG-*Igf1*^KO^ mice and *Igf1*^WT^ controls. Pictures were taken with ×20 magnification. (**G**) Bar graph showing the mRNA expression of astrocyte maker *Gfap* in brains of MG-*Igf1*^KO^ (n = 10) and *Igf1*^WT^ mice (n = 10). Here, 18S was used to normalize data, and animals were pooled from 3 independent experiments. For the comparison of the two groups, the Mann–Whitney U nonparametric test was used, and data are represented as mean ± SEM. Asterisks indicate significant differences (* *p* < 0.05).

Microglia are the major source of IGF-1 in the brain during postnatal development [6], and together with other glia in the CNS, they react to IGF-1 via IGF-1R [11]. We have performed histology and gene expression analysis to investigate the role of microglial-*Igf1* for microglia and astrocyte modulation. Interestingly, IBA-1 staining (Figure 4A) and stereology analysis (Figure 4B) revealed elevated numbers of microglia in MG-*Igf1*^KO^ compared to *Igf1*-intact control. Moreover, we found the upregulation of microglial genes *Sall1*, *Aif1* (gene encoding IBA-1), and *Trem2* in MG-*Igf1*^KO^ compared to *Igf1*-intact control brain tissue, further confirming an increase in microglia (Figure 4C–E). We have observed no changes in astrocytic GFAP protein and gene expression or astrocyte numbers (Figure 4F,G).

### 3.4. Postnatal Deficiency of Microglia-Derived Igf1 Leads to Pronounced Myelination Deficits

**Figure 5:**
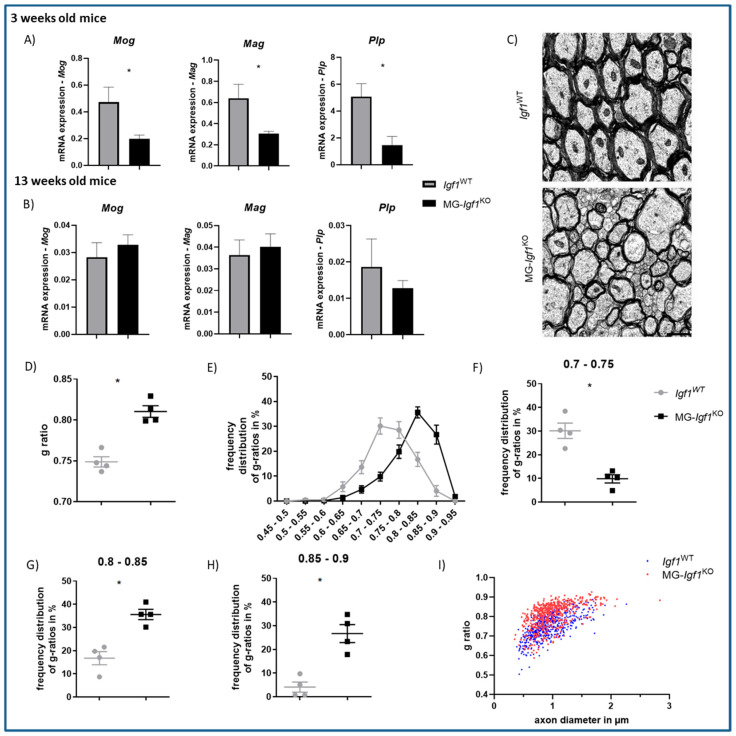
Impact of microglial-*Igf1* deficiency on primary myelination. (**A**) Bar graphs showing mRNA expression of *Mog*, *Mag*, and *Plp* in 3-week-old MG-*Igf1*^KO^ (n = 7) mice and control *Igf1*^WT^ mice (n = 8). (**B**) Bar graphs showing mRNA expression of *Mog*, *Mag*, and *Plp* in 13-week-old MG-*Igf1*^KO^ mice and *Igf1*^WT^ controls (n = 10, 16 for *Mog* and *Mag*, n = 5, 8 for *Plp*). Here, 18S was used to normalize data, and animals were pooled from 3 independent experiments. (**C**–**I**) Electron microscopy analysis of corpus callosum from *Igf1*^WT^ (n = 4) and MG-*Igf1*^KO^ (n = 4) 13-week-old mice. Representative electron microscopy micrographs (**C**), mean G-ratios (**D**), and distribution of G-ratios. (**E**–**H**) Scatter plot showing G-ratios of axonal caliber (**I**). Scale bar = 1 μm. For comparison of the two groups, either an unpaired *t*-test with Welch’s correction was used to analyze normally distributed data, or the Mann–Whitney U nonparametric test was used for data not passing the normality test. Data are represented as mean ± SEM. Asterisks indicate significant differences (* *p* < 0.05).

Given our previous findings demonstrating that a reduction in microglial *Igf1* delays primary myelination in the brain [6], we were interested with respect to whether a nearly complete depletion in microglial-*Igf1* would induce permanent myelination deficits. We initially examined the expression of genes responsible for encoding myelin proteins in brain tissue from 3-week-old animals. Similarly to microglial *Igf1* knockdown [6], we observed a significant downregulation of *Mog*, *Mag*, and *Plp* in MG-*Igf1*^KO^ compared to the controls (Figure 5A). In adult animals, however, we noted no discernible difference in the expression of these myelin-related genes (Figure 5B). Despite the absence of alterations in myelin gene expression in adult animals, we observed striking ultrastructural deficits in myelinated axons within the corpus callosum (Figure 5C), as evidenced by a significantly higher G-ratio (Figure 5D). Conversely, we noted an increased representation of less myelinated fibers (G-ratio: 0.8–0.85; 0.85–0.9) and significantly fewer sufficiently myelinated axons (G-ratio = 0.7–0.75) (Figure 5E–H) in the corpus callosum of MG-*Igf1*^KO^ compared to controls. In addition, when the g-ratio was plotted against the axonal caliber [26] the shift toward a higher G-ration was evident in MG-*Igf1*^KO^, especially for axons with a diameter of ≤1 μm (Figure 5I).

## 4. Discussion

Here, we present a mouse model MG-*Igf1*^KO^ wherein *Igf1* was conditionally removed from microglia. This near-complete ablation of *Igf1* from microglia during the critical first postnatal week, a period characterized by peak microglial-*Igf1* expression, resulted in significant impairments in both cerebral and somatic growth along with notable behavioral alterations. Intriguingly, this *Igf1* deficiency led to increased microglia density within the brain and disturbed primary myelination. These findings identify the importance of microglia-derived IGF-1 in facilitating developmental processes and ensuring proper brain function.

It is well established that the disruptions of IGF-1 signaling exert a profound impact on both somatic and cerebral growth [27]. Global IGF-1 or its receptor (IGF-1R) deficiency typically leads to lethality, and in the case of survival, individuals present with microcephaly and suppressed somatic growth [11]. Similarly, the targeted depletion of IGF-1R in Nestin^+^ cells results in brain and somatic growth retardation, underscoring the pivotal role of neurons as mediators of IGF-1 signaling [28]. Our prior research has demonstrated that partial *Igf1* depletion in postnatal microglia leads to a decrease in brain weight without impact on somatic growth [6]. In the current study, we provide evidence that nearly complete *Igf1* deficiency in microglia leads to a more pronounced effect, with a significant reduction in cerebral and somatic growth. In contrast, the partial conditional deletion of *Igf-1r* from microglia and border-associated macrophages in older mice has led to an increase in body weight [29]. One could speculate that this is possibly due to increased IGF-1 availability for other brain cells. Altogether, these data suggest microglia as important regulators of IGF-1 bioavailability for controlling the somatotropic axis.

A deficiency of both IGF-1 and its receptor has been linked to hypomyelination [11] with the reduced expression of myelin-specific proteins and a decrease in the number of oligodendrocytes during brain development [8]. In contrast, the transgenic overexpression of *Igf1* has been shown to result in an increase in oligodendrocytes and enhanced myelin production [30]. In our previous work, we demonstrated that *Igf1* expression in the CD11c+ microglial subset plays a critical role in directing myelination via *Igf1* expression [6]. The partial deletion of *Igf1* in this subset led to a significant reduction in the expression of genes associated with myelin, as well as myelin proteins, ultimately affecting myelin ultrastructure in 3-week-old mice. These changes were, however, transient, and normally appearing myelin was formed at a later time point [6]. Similarly, our current findings reveal a significant decrease in myelin-associated genes, such as *Plp*, *Mag*, and *Mog* in 3-week-old MG-*Igf1*^KO^. Interestingly, although deficits in myelin gene or protein expression appear to be transient, the significant structural myelin deficits persist to adulthood, underlying the critical role of microglia in driving primary myelination.

Microglia not only express *Igf1*, but they also respond to it via IGF-1R. Ivan et al. demonstrated that the targeted deletion of the IGF-1 receptor specifically from microglia results in morphological changes and minor alterations in microglial transcriptomes [29]. In our study, we show that deficiency in microglial IGF-1 leads to an increase in microglia numbers within the brain. In contrast, Ivan et al. reported no abrogation in microglia density, which may be attributed to the partial IGF-1R deletion in microglia in their study. Nevertheless, these findings strongly suggest the existence of an autocrine IGF-1 signaling pathway in microglia, which plays an important role in regulating both their function and population.

In addition to structural, molecular, and cellular abnormalities, the deletion of *Igf1* from microglia also resulted in notable behavioral anomalies. While we did not observe any deficits in exploratory behavior, such as the number of rearing events in the open field, MG-*Igf1*^KO^ exhibited a pronounced fear response toward novel objects. They displayed minimal interest in exploring novel objects or engaging in marble burying, instead showing freezing behavior that is indicative of neophobia throughout tests. Conversely, IGF-1 has been found to modulate the extinction of the fear memory, and treatment with IGF-1 resulted in a suppressed fear response [15]. This implies a crucial role for microglia in regulating fear responses via the expression of *Igf1*. Notably, anxiety and phobias are common in children with autism spectrum disorders (ASDs), and it has been shown that the levels of IGF-1 in cerebrospinal fluid in children with ASD are significantly reduced [31,32]. Repetitive behavior is another characteristic symptom of ASD. Similarly, our study revealed the increased mean duration of grooming behavior, suggesting either a self-soothing mechanism or repetitive behavior [22,33]. Furthermore, microglial dysregulation is a prominent factor in the development of ASD [34]. These findings collectively suggest that microglial dysfunction leading to the suppression of *Igf1* expression may contribute to the development of psychiatric disorders such as ASD.

Taken together, our data unveil a novel and critical role of microglia as significant contributors to the development and regulation of cerebral and somatic growth and the induction of myelination and proper brain function.

## Figures and Tables

**Table 1:** Schematic overview of used TaqMan™ Gene Expression Assays. The table illustrates a schematic overview of the used primers, their targets, and individual assay ID.

Primer	Target	Assay ID
*Igf1*	Insulin-like growth factor 1	Mm00439560_m1
*Mog*	Myelin oligodendrocyte glycoprotein	Mm00447824_m1
*Mag*	Myelin-associated glycoprotein	Mm00487538_m1
*Plp*	Myelin proteolipid protein	Mm01297210_m1
*Gfap*	Glial fibrillary acidic protein	Mm01253033_m1
*Sall1*	Sall-like Protein 1	Mm00491266_m1
*Aif1*	Allograft inflammatory factor 1	Mm00479862_g1
*Trem2*	Triggering receptor expressed on myeloid cells 2	Mm04209424_g1
*18S*	18S ribosomal RNA	00853255

## Data Availability

The data will be available upon request.

## References

[1] Benmamar-Badel A., Owens T., Wlodarczyk A. (2020). Protective Microglial Subset in Development, Aging, and Disease: Lessons From Transcriptomic Studies. Front. Immunol..

[2] Ginhoux F., Greter M., Leboeuf M., Nandi S., See P., Gokhan S., Mehler M.F., Conway S.J., Ng L.G., Stanley E.R. (2010). Fate mapping analysis reveals that adult microglia derive from primitive macrophages. Science.

[3] Ginhoux F., Prinz M. (2015). Origin of microglia: Current concepts and past controversies. Cold Spring Harb. Perspect. Biol..

[4] Elmore M.R., Najafi A.R., Koike M.A., Dagher N.N., Spangenberg E.E., Rice R.A., Kitazawa M., Matusow B., Nguyen H., West B.L. (2014). Colony-stimulating factor 1 receptor signaling is necessary for microglia viability, unmasking a microglia progenitor cell in the adult brain. Neuron.

[5] Paolicelli R.C., Bolasco G., Pagani F., Maggi L., Scianni M., Panzanelli P., Giustetto M., Ferreira T.A., Guiducci E., Dumas L. (2011). Synaptic pruning by microglia is necessary for normal brain development. Science.

[6] Wlodarczyk A., Holtman I.R., Krueger M., Yogev N., Bruttger J., Khorooshi R., Benmamar-Badel A., de Boer-Bergsma J.J., Martin N.A., Karram K. (2017). A novel microglial subset plays a key role in myelinogenesis in developing brain. EMBO J..

[7] Kettenmann H., Kirchhoff F., Verkhratsky A. (2013). Microglia: New roles for the synaptic stripper. Neuron.

[8] Ye P., Li L., Richards R.G., DiAugustine R.P., D’Ercole A.J. (2002). Myelination is altered in insulin-like growth factor-I null mutant mice. J. Neurosci..

[9] Ueno M., Fujita Y., Tanaka T., Nakamura Y., Kikuta J., Ishii M., Yamashita T. (2013). Layer V cortical neurons require microglial support for survival during postnatal development. Nat. Neurosci..

[10] Netchine I., Azzi S., Le Bouc Y., Savage M.O. (2011). IGF1 molecular anomalies demonstrate its critical role in fetal, postnatal growth and brain development. Best Pract. Res. Clin. Endocrinol. Metab..

[11] Beck K.D., Powell-Braxton L., Widmer H.R., Valverde J., Hefti F. (1995). Igf1 gene disruption results in reduced brain size, CNS hypomyelination, and loss of hippocampal granule and striatal parvalbumin-containing neurons. Neuron.

[12] Laron Z. (2001). Insulin-like growth factor 1 (IGF-1): A growth hormone. Mol. Pathol..

[13] Wrigley S., Arafa D., Tropea D. (2017). Insulin-Like Growth Factor 1: At the Crossroads of Brain Development and Aging. Front. Cell Neurosci..

[14] Yona S., Kim K.W., Wolf Y., Mildner A., Varol D., Breker M., Strauss-Ayali D., Viukov S., Guilliams M., Misharin A. (2013). Fate mapping reveals origins and dynamics of monocytes and tissue macrophages under homeostasis. Immunity.

[15] Liu J.L., Grinberg A., Westphal H., Sauer B., Accili D., Karas M., LeRoith D. (1998). Insulin-like growth factor-I affects perinatal lethality and postnatal development in a gene dosage-dependent manner: Manipulation using the Cre/loxP system in transgenic mice. Mol. Endocrinol..

[16] Sahasrabuddhe V., Ghosh H.S. (2022). Cx3Cr1-Cre induction leads to microglial activation and IFN-1 signaling caused by DNA damage in early postnatal brain. Cell Rep..

[17] Lambertsen K.L., Gramsbergen J.B., Sivasaravanaparan M., Ditzel N., Sevelsted-Moller L.M., Olivan-Viguera A., Rabjerg M., Wulff H., Kohler R. (2012). Genetic KCa3.1-deficiency produces locomotor hyperactivity and alterations in cerebral monoamine levels. PLoS ONE.

[18] Antunes M., Biala G. (2012). The novel object recognition memory: Neurobiology, test procedure, and its modifications. Cogn. Process.

[19] Wirenfeldt M., Dalmau I., Finsen B. (2003). Estimation of absolute microglial cell numbers in mouse fascia dentata using unbiased and efficient stereological cell counting principles. Glia.

[20] Himanshu, Dharmila, Sarkar D., Nutan (2020). A Review of Behavioral Tests to Evaluate Different Types of Anxiety and Anti-anxiety Effects. Clin. Psychopharmacol. Neurosci..

[21] Seibenhener M.L., Wooten M.C. (2015). Use of the Open Field Maze to measure locomotor and anxiety-like behavior in mice. J. Vis. Exp..

[22] Kalueff A.V., Stewart A.M., Song C., Berridge K.C., Graybiel A.M., Fentress J.C. (2016). Neurobiology of rodent self-grooming and its value for translational neuroscience. Nat. Rev. Neurosci..

[23] Kalueff A.V., Tuohimaa P. (2004). Grooming analysis algorithm for neurobehavioural stress research. Brain Res. Brain Res. Protoc..

[24] Leger M., Quiedeville A., Bouet V., Haelewyn B., Boulouard M., Schumann-Bard P., Freret T. (2013). Object recognition test in mice. Nat. Protoc..

[25] Valvassori S.S., Borges C.P., Varela R.B., Bavaresco D.V., Bianchini G., Mariot E., Arent C.O., Resende W.R., Budni J., Quevedo J. (2017). The different effects of lithium and tamoxifen on memory formation and the levels of neurotrophic factors in the brain of male and female rats. Brain Res. Bull..

[26] Begolly S., Shrager P.G., Olschowka J.A., Williams J.P., O’Banion M.K. (2016). Fractionation Spares Mice From Radiation-Induced Reductions in Weight Gain But Does Not Prevent Late Oligodendrocyte Lineage Side Effects. Int. J. Radiat. Oncol. Biol. Phys..

[27] Al-Samerria S., Radovick S. (2021). The Role of Insulin-like Growth Factor-1 (IGF-1) in the Control of Neuroendocrine Regulation of Growth. Cells.

[28] Kappeler L., De Magalhaes Filho C., Dupont J., Leneuve P., Cervera P., Perin L., Loudes C., Blaise A., Klein R., Epelbaum J. (2008). Brain IGF-1 receptors control mammalian growth and lifespan through a neuroendocrine mechanism. PLoS Biol..

[29] Ivan D.C., Berve K.C., Walthert S., Monaco G., Borst K., Bouillet E., Ferreira F., Lee H., Steudler J., Buch T. (2023). Insulin-like growth factor-1 receptor controls the function of CNS-resident macrophages and their contribution to neuroinflammation. Acta Neuropathol. Commun..

[30] Carson M.J., Behringer R.R., Brinster R.L., McMorris F.A. (1993). Insulin-like growth factor I increases brain growth and central nervous system myelination in transgenic mice. Neuron.

[31] Vanhala R., Turpeinen U., Riikonen R. (2001). Low levels of insulin-like growth factor-I in cerebrospinal fluid in children with autism. Dev. Med. Child. Neurol..

[32] Riikonen R., Makkonen I., Vanhala R., Turpeinen U., Kuikka J., Kokki H. (2006). Cerebrospinal fluid insulin-like growth factors IGF-1 and IGF-2 in infantile autism. Dev. Med. Child. Neurol..

[33] Mu M.D., Geng H.Y., Rong K.L., Peng R.C., Wang S.T., Geng L.T., Qian Z.M., Yung W.H., Ke Y. (2020). A limbic circuitry involved in emotional stress-induced grooming. Nat. Commun..

[34] Hu C., Li H., Li J., Luo X., Hao Y. (2022). Microglia: Synaptic modulator in autism spectrum disorder. Front. Psychiatry.

